# Introducing a single point of access (SPA) to child and adolescent mental health services in England: a mixed-methods observational study

**DOI:** 10.1186/s12913-020-05463-4

**Published:** 2020-07-08

**Authors:** Stephen Rocks, Margaret Glogowska, Melissa Stepney, Apostolos Tsiachristas, Mina Fazel

**Affiliations:** 1 Health Economics Research Centre, Nuffield Department of Population Health, University of Oxford, Richard Doll Building, Old Road Campus, Oxford, OX3 7LF England; 2 The Health Foundation, 8 Salisbury Square, London, EC4Y 8AP England; 3 Nuffield Department of Primary Care Health Sciences, University of Oxford, Radcliffe Primary Care Building, Radcliffe Observatory Quarter, Woodstock Rd, Oxford, OX2 6GG England; 4 Department of Psychiatry, University of Oxford, Warneford Hospital, Oxford, OX3 7JX England

**Keywords:** Child and adolescent psychiatry, Mental health services, Service reorganisation, Access to services, Service improvement

## Abstract

**Background:**

In many high-income countries, primary care practitioners are the main point of referral for specialist mental health services. In England, Child and Adolescent Mental Health Services (CAMHS) are increasingly adopting a Single Point of Access (SPA) to streamline referrals and introduce self and parent/carer-referrals. This involves a significant shift of responsibility from primary care towards CAMHS who adopt a more active role as gatekeeper for their service. This study evaluates the adoption of a SPA in CAMHS across a large region in England.

**Methods:**

We conducted an observational mixed methods study in two CAMHS from January 2018 to March 2019 to evaluate the adoption of a SPA. We collected quantitative data from electronic patient records and qualitative data through ethnographic observation and in-depth interviews of staff and stakeholders with experience of using CAMHS. Additional data on volumes was shared directly from the SPAs and a further snapshot of 1 week’s users was collected.

**Results:**

A similar SPA model emerged across the two services. Staff were positive about what the model could achieve and access rates grew quickly following awareness-raising activities. Despite the initial focus being on a telephone line, online referrals became the more regularly used referral method. Increased access brought challenges in terms of resourcing, including identifying the right staff for the role of call handlers. A further challenge was to impose consistency on triage decisions, which required structured information collection during the assessment process. Similar to GP referrals, those self-referring via the SPA were mainly from the least deprived areas.

**Conclusions:**

The introduction of a SPA has the potential to improve young people’s access to mental health services. By addressing some of the barriers to access, simplifying where to go to get help and making it easier to contact the service directly, a SPA can help more individuals and families access timely support. However, the introduction of a SPA does not in itself expand the capacity of CAMHS, and therefore expectations within services and across sectors need to be tempered accordingly. SPA services providing different referral approaches can further improve access for the harder to reach populations.

## Background

A significant number of young people experience a mental health disorder [1, 2]. A 2017 survey in the UK found that almost 13% of 5 to 19 year olds had at least one mental disorder, with evidence of rates increasing with age [3]. Untreated mental health conditions negatively impact on development throughout the life course [4]. However, although effective interventions exist [5], most people do not receive treatment in a timely manner [6, 7]. For example It is estimated that less than a quarter of young people with a mental health problem receive any help from specialist services [7], despite evidence that timely interventions can improve longer-term outcomes [5].

Research has identified a number of structural barriers to accessing child and adolescent mental health services (CAMHS) that can interplay with stigma and fear to the detriment of the young people with needs. Barriers exist for potential referrers, including for parents/carers, GPs, other professionals in education and social care as well as for young people themselves [8]. This can be exacerbated by a lack of awareness of existing services and pathways of care, especially when services change as dramatically as they have done in the UK [9]. Often, the parent/carer needs to identify a difficulty in their child as well as have a willingness to take them to health services. This can then be compounded by the primary care worker needing to decide to refer on, which can entail a number of further barriers before any direct discussion about a young person has taken place with CAMHS [10, 11]. In addition, the process of connecting with services can be time-consuming and drawn out, which may be a further deterrent [8]. It is common for parents to discuss difficulties with the education sector [12], but many teachers express concern that they lack the skills to identify which mental health problems might warrant onward referral [13].

There are a number of key models to explain how mental health care is accessed by young people [14]. In many countries, with a focus on individual drivers to accessing care, primary care is the main point of referral to specialist mental health services, often through general/family practitioners (GPs). Young people with a mental health disorder are often in contact with primary care but might present with a range of difficulties, not necessarily overly psychological, but with pain or headaches, for example [15]. A study in the UK found that GPs only identify and refer a minority of cases with mental health problems to specialist services, although this increased when parents expressed concern to the GP [16]. Possible reasons for the low referral rate among GPs are a lack of confidence, skills and knowledge in identifying mental health problems [10, 17]. GPs may also perceive limited availability within specialist services with reports of long waiting times to first appointments, and may determine that the difficulties are likely to respond better through alternative strategies, either in primary care or through other resources and services [10]. This is mirrored in other models of ‘gateway’ provision of services, where referrals to mental health services for those with mental health needs can be low if those who are first accessed have limited knowledge of mental health presentations and needs [14]. Barriers may also concern young people more directly. Young people may not want or may not be able to involve key adults in the process of referral or treatment [9, 18], such as their parents, and may be concerned about how their difficulties might be perceived by other professionals [9]; furthermore, they might not appreciate that their symptoms have a psychological origin. Research suggests the ability for young people to self-refer could help overcome these barriers [19–22].

Following the 2015 Government review, *Future in Mind* [23], and other directives, such as the *Five Year Forward View For Mental Health* [24]*,* CAMHS in England were tasked by NHS England with submitting ‘transformation’ plans. These needed to set out how the health system would work together and with other agencies to achieve the aims of: building resilience; improving accessibility, especially for more vulnerable patients; and improving patient experience [23]. *Future in Mind* specifically cited a Single Point of Access (SPA) as a way to achieve greater clarity over where people should go to seek help. It identified the following features of a SPA: a) a single point of contact for a range of universal services covering advice, consultation, assessment and onward referral; b) early risk assessment; c) prompt decision-making about which team can best meet the child/young person’s needs; and d) the ability for young people and parents to self-refer [23]. These goals align with changes that have been identified to improve access in Canada, Australia, Ireland and the US [25–28]. For example, the changes follow models of improving provision of mental health care by altering provider behaviour and in this model, the provider becomes the mental health service itself, and so brings mental health expertise closer to young people’s initial point of contact in the hope that this will improve the quality of care decisions [14].

The introduction of a SPA has subsequently become a common component of many CAMHS ‘transformation plans’ across England and involves a significant shift of responsibility within the health system towards CAMHS, which would seek to streamline access to the service and adopt a more active role as gatekeeper. With many CAMHS transformations underway, evaluation of this specific component is critical to learn how SPA is being adopted, to understand the implications of this shift and to inform the roll-out of these services across England. In this observational, mixed methods study, we evaluate the adoption, implementation and delivery of a the SPA in two CAMHS, as part of a larger evaluation study [29].

## Methods

### Study setting

The study was conducted in South East England and included CAMHS provided by Oxford Health NHS Foundation Trust (Oxford Health), one of the largest CAMHS providers in England [30]. We investigated the adoption of SPA as part of the CAMHS transformations in Buckinghamshire and Oxfordshire.

### Design and research methods

Qualitative data collection (undertaken by postdoctoral researcher, MS, between January 2018 and March 2019) included 80 h of ethnographic observations: shadowing key staff; informal interviews with different stakeholders; and attending a range of team meetings. In addition, in-depth interviews were conducted (*n* = 30). These comprised of eighteen in-depth interviews of staff in both Oxfordshire and Buckinghamshire with administrative and clinical staff, including those involved in the SPA, specialist services, third sector partners, and service managers (see Supplementary file for Interview Guides). Interviews were also carried out with eight young people and four parents/carers (2 paired interviews) with experience of using CAMHS both before and after the transformation. We used early interviews to inform our approach to sampling and identifying further participants for interview, in order to capture as wide a range of experiences among the workforce as possible. Young people and their carers/family were recruited through advertisements. Topic guides were developed, informed by the literature, clinical experience within the team and the ongoing ethnographic observations.

All the interviews were digitally recorded and professionally transcribed. The analysis of the ethnographic field notes and two sets of interviews proceeded utilising the following method: first the field notes and transcripts were read and re-read; initial ideas for themes were noted; and systematic and detailed open coding was then conducted using NVivo 12 [31], a qualitative data analysis software package which assists in the organisation and retrieval of data. The coding of the first set of field notes and interviews with staff generated an initial coding framework and the interviews with young people another coding framework, both of which were discussed by MS and MG. These were further developed and refined as data collection and analysis proceeded and developed further with the research team. The research team also critically discussed ideas for categories and themes emerging from the data, to ensure trustworthiness.

Data from electronic patient records was available from April 2012 to March 2019. Data is recorded for referrals to and contacts with CAMHS. This includes demographic information for the patient, detail of contacts undertaken and information regarding the referral source. The observation period for this study included a pre- and post-transformation period. Not every contact made to the SPA is recorded on the electronic patient record system, for example if a parent is phoning to ask information on when their next appointment might be. Additional data on volumes was shared directly from the SPA in Buckinghamshire and Oxfordshire and a snapshot of 1 week’s users of SPA was collected from February 11-15th 2019 (Oxfordshire) and March 11-15th 2019 (Buckinghamshire). Descriptive analysis was performed to understand the breakdown of those using the SPA. Statistical tests, t-test or chi-squared, were used to determine whether those using SPA differed from those referred by GPs [32]. All analysis was performed in Stata 14.

## Results

### Intervention

A SPA was introduced in Buckinghamshire in 2015/16 and in Oxford in 2018/19. In both services, staff perceived the introduction of the SPA as one of the main components of the overall service transformation taking place. The adoption, implementation and delivery of the SPA are described below following the themes identified from analysis of the ethnographic and interview data with quantitative data providing triangulation of the findings.

Both SPA services involved a central team based in one location taking calls and online referrals from health, social care and education professionals, third sector workers, parents and families, as well as young people themselves. Staff use the information gathered from telephone consultations, and other referral and questionnaire data to decide on the appropriate next step which can include gathering further information, signposting or a referral to CAMHS. Within CAMHS there are different pathways, with various specialist teams offering different levels of support, such as the neurodevelopmental pathway for disorders including those on the Autism Spectrum. There are broad similarities in the staffing and organisation of the SPA services, but also some differences.

### How to make contact with the SPA

In both SPA services, non-clinical staff answer direct calls and collect basic information and for online referrals some structured questions need to be answered. In Buckinghamshire the majority of requests for service will result in a clinician call whilst in Oxfordshire the clinicians will review the information and then make a decision about whether to call the individual back, gather more information or send information about resources including self help materials. If the request is more appropriate for another service, the SPA will advise the caller to contact other services or, with their consent, will make a referral for them to other statutory or third sector agencies.

In Oxfordshire, the SPA is fully staffed by Oxford Health CAMHS; the initial call being taken by an administrator. The SPA system in Buckinghamshire was set up with the involvement of Contact Support Workers (CSWs) from a third sector organisation, Barnardo’s, which is a relatively large children’s charity working to support vulnerable children through specialist workers and policy advocacy. In this SPA, CAMHS provide clinical input and oversight to the CSWs. The involvement of third sector workers caused some initial anxiety, for example CAMHS staff expressed concern that the CSWs might be asked to provide support beyond their specified, administrative role and might also find it difficult to manage some of the more aggressive or delicate interactions with distressed callers. This was in the context of a larger process of building familiarity and trust between organisations. A number of staff members shared reservations they had felt at the time:

*“It felt like [third sector workers] were coming into Oxford Health as opposed to us coming in together. It's not an issue now.” (Staff interview)*

While much of the emphasis was initially placed on the phone line; as increasing referrals came through online routes, the online referral forms needed some revision to ensure sufficient information was gathered for a triage decision to take place:

*“….it was just kind of a box and just said, ‘what's the problem?’ They could just put in, I mean, my son is angry and then we would then have to ring because we need more information than that.” (Staff interview)*

### Access rates

It was clear from all staff that they perceived volumes of contacts were rising.

*“It's good, because we are getting more referrals in and we are more accessible”*

*(Staff interview,)*

*“We know from the data coming in, the numbers coming in through SPA we used to get…30 to 40 referrals a day across the county. We are now getting 50 to 70 written referrals a day across the county and between 10 and 25 telephone calls a day on top of those. It's anywhere between 60 and 90 to 100 referrals a day.” (Staff interview)*

**Fig. 1:**
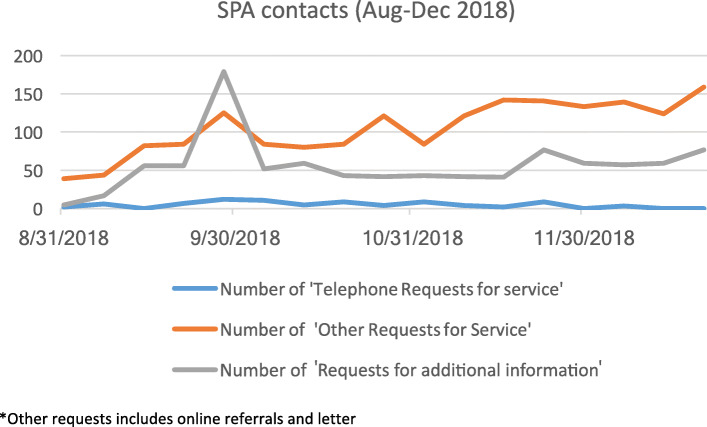
SPA contacts in Oxfordshire August – December 2018

Quantitative data supports this. A snapshot of data from the SPA shows the number of requests for service in Oxfordshire rose from 40 per day to 160 between August and December 2018 (Fig. 1). Online referral in Buckinghamshire SPA was introduced after the main service had started and proved a popular addition:

*“because I think one of the really good things has been the online referral. Many, many more people use the online—massively.” (Staff interview)*

In Oxfordshire, the majority of requests, by the end of the study period, were from the online portal (captured as ‘other’ in Fig. 1). This was reflected in positive views of parents and young people towards an online service.

*“(Family member) Because […] the bridge of talking to someone, the step of going and doing it online, it's not face-to-face. It's just one less hurdle really, isn't it?” (Parent and child interview)*

**Fig. 2:**
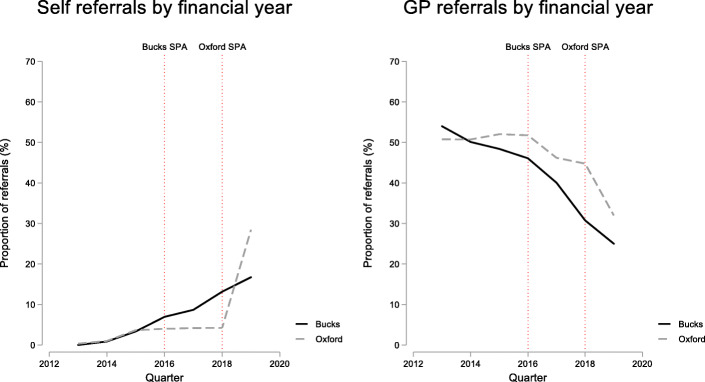
Proportion of self and GP referrals following launch of SPA services

The impact of SPA was also visible in data from the electronic patient record (EPR), which covers those with a referral to specialist services. While different groups can use SPA, the most marked change observed was in an increase in the proportion of self-referrals by young people, parents and carers. This increased steadily in Buckinghamshire following transformation to around 15% of referrals in 2018/19 (Fig. 2). The increase in Oxfordshire was even more abrupt, rising from around 5% to more than 25% in the same year. The increase in self-referrals was matched by a decline in the proportion of referrals coming from GPs either because GPs had advised families to call directly or because GPs were not involved in the decision to refer, which might have come from school or social care.

### Who accesses SPA

Data from the SPA suggest users come from a wide range of backgrounds, with professionals, parents/carers and young people utilising the new service. A snapshot of the phone line shows that the biggest single group of users in Buckinghamshire and Oxfordshire were parents (69 and 50%, respectively – see Table 1), but teachers, professionals from social care, young people and GPs also contact the service. A significant minority of calls in both services were for more information about existing referrals (35% in Buckinghamshire). A majority of calls (52%) in Buckinghamshire were for young people with low-mood and anxiety (Table 2).

**Table 1:** Snapshot of SPA - who contacts

	Buckinghamshire	%	Oxfordshire	%
Parent	62	69%	51	50%
Teacher	9	10%	14	14%
Social care	4	4%	13	13%
Other health	1	1%	8	8%
Young person	12	13%	5	5%
School nurse/c’sellor			4	4%
GP			4	4%
Other	2	2%	3	3%
	90		102

**Table 2:** Snapshot of SPA - reason for contacting (Buckinghamshire only)

Reason for calling	No.	%
Information on care of Young Person	23	26%
Low mood	23	26%
Anxiety	16	18%
Suicidal ideation	10	11%
Autism Spectrum Disorder	11	12%
Sleep	4	4%
Other (safeguarding, psychosis, eating problems)	3	3%

**Fig. 3:**
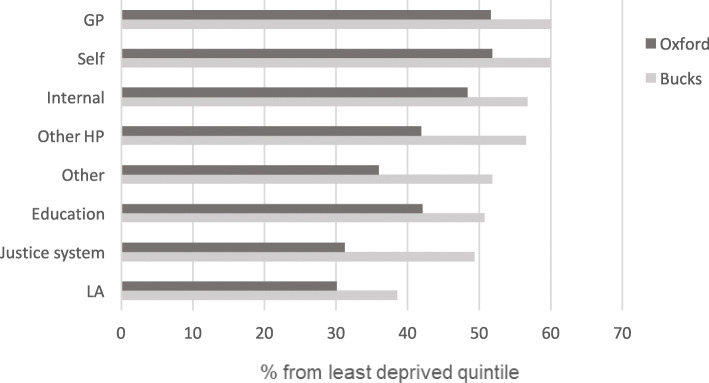
Proportion of referrals from least deprived quintile, by source

A particular innovation of CAMHS is self-referrals, both those calls directly from parents/carers or young people themselves (primarily aged 16–17, although the service will talk to any child that calls) who can contact CAMHS directly. Looking in more detail at the characteristics of self- referrals, data from the respective services post-transformation shows that the majority of self-referrals are for children/young people living in the least deprived quintile nationally according to the index of multiple deprivation (60% in Buckinghamshire and 52% in Oxfordshire). This is similar to the pre-transformation rate for referrals from GPs (Fig. 3).

Table 3 shows a range of demographic characteristics of the child or young person referred. This includes those referred by the GP 1 year before the introduction of the SPA, taken to be the main route into the service at that point. Following transformation (2015/16 in Buckinghamshire, 2018/19 in Oxfordshire), nearly all referrals come through the SPA but the source varies. Table 3 also shows the characteristics of those referred by the GP and those self-referred in the period following the introduction of the SPA with a comparison between the two groups. In Buckinghamshire, the only significant difference is in ethnicity, with those self-referring more likely to be White British. In Oxfordshire, while the proportion coming from the least deprived quintile is similar to GP referrals (Fig. 3), on average those who self-referred are from less deprived areas as well as being younger and a higher proportion are White British.

**Table 3:** Characteristics of children referred by GP or self-referred

	1 year before SPA introduction	Following introduction of SPA		Difference
	(1) GP	(2) GP	(3) Self	(3)–(2)
Buckinghamshire				
Ward deprivation (IMD)	10.3	10.4	10.5	0.1
Average age (years)	12.6	12.5	12.6	0.0
% Male	46.7	46.1	48.0	1.9
% White British	84.5	81.9	84.1	2.2^a^
Oxfordshire				
Ward deprivation (IMD)	11.7	11.8	11.1	−0.7^a^
Average age (years)	12.0	12.4	11.3	−1.0^a^
% Male	48.9	50.2	50.3	0.1
% White British	85.0	55.2	84.0	28.8^a^

Note: ^a^means significant difference at the 95% confidence level; t-test for continuous, chi-square for categorical variables

Buckinghamshire records every request for service, whether or not that request is accepted. Of 2953 self-referrals received in Buckinghamshire, 765 (26%) were deemed not in need of specialist CAMHS referral and were given advice on how to manage the difficulties or to contact other services. A high proportion of self-referrals were accepted into specialist CAMHS (74.1%), with slightly lower proportions of referrals accepted from education (70.5%) and GPs (68.5%) (Table 4).

**Table 4:** Percentage of accepted referrals in Bucks by referrer characteristics

Referrers to Bucks CAMHS	
Source	Accepted or waiting (%)
GP	68.5
Education	70.5
Self	74.1
Local authority	77.3
Other (including third sector workers)	77.6
Other health professional	84.1
Justice system	96.2
Internal	99.4

Self-referrals that did not get referred into CAMHS were more likely to be for children or young people that were male, younger, more deprived and non-White-British. Only the difference in ethnicity was not statistically significant at the 95% level (Table 5).

**Table 5:** Characteristics of self-referrals: accepted vs. rejected

	(1) Accepted or waiting	(2) Rejected	Difference (2–1)
Ward deprivation (IMD)	10.3	11.1	0.8^a^
Average age (years)	12.8	12.0	−0.8^a^
% Male	46.9	51.1	4.1^a^
% White British	84.9	81.9	−2.9

Note: ^a^means significant difference at the 95% confidence level; t-test for continuous, chi-square for categorical variables

### The advantages of having a SPA

Staff agreed that the purpose of the SPA was to improve access by having a single point of contact available to different groups:

*“…anyone can access us and have a conversation as soon as they have got a question about mental health and we will listen to them and try and do something with that.” (Staff interview)*

*“It's fantastic that we are being able to do it over the phone…It's helping like the more like risky or needy children, children with more complex needs get seen quicker…” (Staff interview)*

Staff confirmed that not all requests for service enter specialist CAMHS, but they reported feeling able to offer some support. For one, the very fact that callers could speak to someone in SPA could itself be a source of reassurance. Although staff could signpost to alternative help or support, this signposting was not always viewed as supportive by callers.

Of note, the process was considered quicker and less demanding for parents, young people and professionals than under the previous system where longer referral forms had to be completed by the referrers (usually GPs).

*“You don't have to tell your GP and tell your parents who is going to tell the school who is going to tell x, y, z.” (Young person interview)*

*“It's not necessarily thinking, I [GP] am going to have to type up a referral form and then I am not sure if this is going to meet the criteria. They know that they can just call up and run a like no-name consultation sort of thing through the SPA to sort of say, is it worth doing a referral into the service or is it not?” (Staff interview)*

The SPA gives a consistent point of contact, which callers can use to check the status of a referral - a significant number of calls were for additional information, such as confirming the location of an appointment, rather than a new request for service. Staff suggested a caller could also use the SPA to report if a situation had deteriorated and to ask to have the referral expedited. Finally, staff reported that SPA simplified the re-referral process:

*“…you can ring in at any time and you can just be reopened. You don't need a letter. You don't need a form. You don't need anybody else to do it for you. I think that's a big plus.” (Staff interview)*

In addition, interviews and ethnography revealed examples of speedy responses, such as a school nurse being able to easily contact SPA and refer an urgent case. Staff reported receiving positive feedback from schools and GPs regarding the new service.

### The challenges of SPA

The two main challenges that emerged were capacity and consistency. Firstly, the SPA seemed to become a victim of its own success because it had increased numbers calling without the capacity to manage. For instance, the initial aim was for the SPA to respond to all calls with a decision within 3 days, however as activity ramped up this proved difficult. A number of staff reported working extra hours to deal with workload and reduce the ‘backlog’ of calls, as this excerpt demonstrates:

*“We are meant to do two days a week in SPA, but because we've had so many calls…If somebody else is on it and I know they're inundated, I'll say ‘I’ll do some of them’…And definitely probably work an extra hour at least each day, and don't take a lunch.” (Staff interview)*

In Oxfordshire, there was an ambition to bring the time for responding to calls down to 1 day. This was not possible during the study period. Staff in Buckinghamshire reflected that the pressure of working in the SPA had resulted in a high turnover of staff, as illustrated by this staff member:

*“And then being in SPA was quite hectic…you have got to be there at eight and leave by six and it's constant… I have seen a lot of people who, you know, people who are leaving.” (Staff interview)*

A second broad challenge was in ensuring that consistent triage decisions were taken. There were mixed views on consistency. Some staff highlighted risks, in particular, the lack of a structured approach when the system was new:

*“…we need some kind of structure that we are all saying the same thing. A parent could call back in the next day and speak to somebody different and be told something completely different.” (Staff interview)*

On the other hand, with a smaller group of people, working closely on the referral process, some staff thought SPA could result in greater consistency in terms of triaging decisions:

*“…it's all getting judged by the same, basically the same four people and so the decisions are all quite similar, which I think is quite good, because different teams, like I said before, about ideas around risk and what is risky to someone is not risky to someone else.” (Staff interview)*

Staff recognised the importance of attaining sufficient information to allow an appropriate triaging decision. An added complexity was that different pathways - the specialist teams who assess and provide treatment for accepted referrals - required different information before accepting them for treatment, as this staff member explained:

*“They (the pathways) do want subtly different things from SPA, different information and different work up. Getting More Help (one pathway) you can put a case straight through. Eating disorders (another pathway) you need to do a lot of ground work first, which is okay, but it's about knowing just that, what they need and what you need and what is doable.” (Staff interview)*

### Barriers and enablers

#### Awareness

Both areas found that it took time for potential users to know about SPA and that they could use it to directly access CAMHS. Telephone survey comments highlighted that some parents had not heard of SPA and that the new system was not – at that point in time - explained on the website or social media platforms. Word of mouth and communication with local partner agencies, including GPs and schools nurses, were considered important in raising awareness, as this excerpt shows:

*“We went and did the school health nurses conference and got all the school health nurses in one hit, which was I think a real tidal change, particularly in phone calls.” (Staff interview)*

In Buckinghamshire, staff reported that it was difficult to convince everyone that a referral did not have to come from a GP. Instead, the belief that a GP referral was a prerequisite for contacting CAMHS persisted for many.

#### Consent, recording and rejected referrals

In the Buckinghamshire SPA calls were immediately logged as referrals. In Oxfordshire, this was not the case. Staff expressed concern that callers would not want a mental health record opened when they may only receive advice and support rather than a referral to specialist services. As a result, Buckinghamshire CAMHS records referrals that are not accepted into specialist CAMHS, whereas for Oxfordshire those who contact the SPA and do not enter the service are not formally recorded on the EPR although it was important to the service that they had not been ‘rejected’, and could get back in touch.

#### Getting staffing right

As SPA was a new service, it brought different demands and required a particular skillset with an onus on processes and communication. The description of the SPA as a ‘call centre’ in one area led to some recruitment difficulties as staff feared a loss of clinical skills. However, in practice some staff found the SPA gave them an opportunity to ‘do more’:

*“…you're having to think on your feet so much quicker. I'm speaking to - you know, doing ten consultations a day rather than four sessions. So you're actually doing quite a lot more, in that sense.” (Staff interview)*

Staff recognised the need to recruit the right people to SPA, but also that those in the role required support. Taking a constant stream of referrals was reported to be emotionally draining, especially as some of the calls could be longer and more demanding.

*“We have had a couple of staff that haven't fitted. I think it is a particular role. And I think trying to make it varied helps. I think if you are just doing referrals all day, you can't last doing that...You need good support.” (Staff interview)*

## Discussion

A Single Point of Access (SPA) was introduced in Oxfordshire and Buckinghamshire that was consistent with the principles set out in *Future in Mind –* the UK government’s 2015 vision for children’s service development. This permitted a more streamlined referral process direct to CAMHS rather than via primary care, and included self-referrals. Access increased with awareness raising activities. However, increased demand brought challenges in terms of staffing and resources. In particular, while the SPA can make it easier to engage with CAMHS, it cannot itself increase access elsewhere in the system.

Staff were mostly positive about the principles of the SPA. By resolving some of the confusion over where to go to get help and by making it easier to speak to someone quickly, SPA was seen by staff and users to bring clarity and speed to the referral process, assisting all the likely users. In particular, this could reduce what may be termed ‘pre-waiting times’. Waiting times are measured from the point of referral to the point of contact, but this fails to capture what can be a long time spent getting to the point of referral [33, 34]. Although not precisely quantifiable - there is no data on the period prior to receiving a referral - SPA has the potential to reduce this and therefore expedite access to support.

Awareness-raising activities were important for increasing volumes accessing SPA, especially as primary care referrals were not required. However, the increase in access to SPA resulted in pressure on resources and forced a more realistic appraisal of what the service could achieve. In particular, although the SPA can expedite referrals, it alone cannot increase the numbers of people accessing a service operating near capacity – that is, not without additional resources elsewhere in the system. This resulted in an inherent tension between the mantra of “all can access” and the pressure felt by staff to limit access to specialist CAMHS pathways. As such, some tempering of staff expectations was needed to cope with the high demand. Future research could also look at triaging decisions. The introduction of other telephone triage services, such as NHS 111, has been shown to have had the similar consequence of increasing the number of people seeking care [35].

The SPA also brings new and unfamiliar roles to CAMHS. Staffing can be challenging, especially in the context of wider staffing shortages across health services and the new roles bring particular pressures, with staff fearing burnout. Different staffing mixes were employed by the services; SPA clinicians could also take on other roles, for example in Oxfordshire their jobs were often split between providing a direct school-based ‘InReach’ mental health service and the SPA.

Online referral, despite not being the initial focus of the SPA, emerged as a particularly popular referral route into CAMHS. Young people perceived it as an easier way to get in contact. This necessitated a structured approach to information collection for online referrals to support SPA staff in taking appropriate and consistent triaging decisions. Digital services are increasingly popular across the NHS as a seemingly resource efficient way to reach a greater number of people [36]. Young people may be more likely to prefer digital services [37], albeit this assumption requires further testing [38]. Online referral transfers the burden of information sharing more completely to the patient/user, there is therefore a risk that inefficiencies might be introduced into the system if it takes longer for SPA staff to gather the necessary information to take the most appropriate triaging decision, or if users find completing such form difficult.

While SPA can increase access, it is less clear to what extent this can help certain vulnerable groups. The characteristics of those self-referred, a particular innovation of SPA, are broadly similar to those referred via primary care, but the self-referred group are more likely to be ‘White British’ and, in Oxfordshire, to live in a less deprived area. The evidence is not conclusive - White British is the ethnicity found to have the greatest mental health need [3], while Oxfordshire is still early in its implementation of the SPA - but this may imply an inequality in access. Given that mental health problems are more common among young people living in lower income households [3], it may be worth considering how outreach could spur more people from deprived areas to use this aspect of the service at an earlier stage.

Finally, a core part of the SPA offer is the provision of information and links to other organisations where specialist CAMHS is not deemed the most appropriate service. Those not accepted into specialist services in Buckinghamshire were younger and more deprived, which suggests some tension with attempts to help those in need to access support more quickly. To that extent, the success of SPA as a preventative intervention rests in part on the value gained from signposting to other forms of support. More research is needed to explore this with parents, carers and young people.

### Limitations

This study is a snapshot at a point of time. The SPA service was only recently introduced in one of the services and subject to ongoing change. We were able to appraise perceptions of the service and to analyse available electronic patient records, but more data would be required to assess, for instance, the appropriateness of triage decisions taken. This could be facilitated by using Routine Outcome Measures with all SPA users. We did not have data on what those accessing SPA did before arriving at the service, therefore we do not know whether those self-referring contacted their GP first. Again, further research could reveal more about the information seeking journey and the extent to which the SPA may reduce demands on primary care.

## Conclusions

The introduction of a SPA has the potential to improve young people’s access to mental health services. By resolving some of the confusion over where to get help and by making it easier to speak to someone directly in CAMHS, a SPA can help more people access timely support. However, in itself, it cannot expand the capacity of specialist CAMHS. Services might need to incorporate further innovations to ensure the SPA improves access for more populations not traditionally accessing services.

## Supplementary information

**Additional file 1.** Interview Guides for CAMHS professions and Children and Young People.

## Data Availability

Anonymised qualitative data interview transcripts are available from the study authors. The electronic patient data are available from Oxford Health NHS Foundation Trust, but applications must be made directly to Oxford Health to access these data, which were used under license for the current study, and returned to Oxford Health when the study was completed. Other data, not including the electronic patient record, are available from the authors upon reasonable request and with permission of Oxford Health NHS Foundation Trust.

## Abbreviations

CAMHS: Child and adolescent mental health services
CSW: Contact support workers
EPR: Electronic Patient Record
GPs: General practitioners
IMD: Index of Multiple Deprivation
Oxford Health: Oxford Health NHS Foundation Trust
SPA: Single point of Access
