# Supplementary material for: Introducing a single point of access (SPA) to child and adolescent mental health services in England: a mixed-methods observational study
Source: BMC Health Serv Res. 2020 Jul 8;20:623. doi: 10.1186/s12913-020-05463-4 (PMC7346657; doi:10.1186/s12913-020-05463-4)
Supplement: Supplementary file 1 — Additional file 1. Interview Guides for CAMHS professions and Children and Young People. [file 12913_2020_5463_MOESM1_ESM.docx]

# Supplementary files: Interview Guides for CAMHS staff and Children and Young People

## Semi-structured Interview Guide for CAMHS staff

### Role in organisation

- Can you tell me a bit about your role here in CAMHS? What is your main role/tasks that you do? Can you describe typical day/day to day tasks before the changes started (focus on old system - follow up for clarification)
- How did you come to work here? What were your motivations for coming to work here? What have you enjoyed/disliked most about your role so far?

### Changes/transformations (RE-AIM Framework – ‘AIM’ focuses on organisation):

#### Adoption & Implementation:

- Can you describe in your own words what you see as the main changes happening at the moment in CAMHS? Why do you think they happened?/What purpose do you think those changes are for?
- What was your role in these changes?
- What do you see as the main benefits of the changes (**probe about InReach/SPA/self-referrals/voluntary agencies**)? What do you think about changes to create SPA?
  - Oxfordshire: School InReach changes?
- Views about voluntary agency involvement
  - Buckinghamshire: Barnardo’s buddies?
- Can you describe how the transformations were first communicated to you? What happened? Who explained them? How did you feel about this at the time? Do you think this was handled well/could have been handled better? (Implementation)
- What sort of training have you had (if any)? Official training?
- What was the general vibe/feeling about these changes amongst colleagues? How were any issues/questions dealt with?
- How have the changes/transformations been affecting you? Can you give me some examples?
- Have your thoughts/feelings changed at all as time has gone on?

#### Maintenance (What is required for service changes to be maintained REAIM framework):

- Buckinghamshire: Have you seen/witnessed the benefits of the changes? What impact do you think the changes have had on the different pathways and on service activity?
- Projecting into the future, in the next year/5 years how do you see the service changes developing? What do you think will be the benefits of those changes in years to come? What impact do you think the changes have had/will have on the different pathways and on service activity?
- Have you had any feedback from service users – CYP and parents/families about the changes? What do you anticipate they will make of the changes?
- Regarding SPA, how often do cases not get referred/you give advice yourself? How do you handle this?
- What criticisms/challenges of the new changes do you anticipate/have you experienced?
- What things do you think service users will appreciate/like?
- What would you say are the things that make it easier for you to better do your job/ manage these changes (facilitators)? Given these changes had to take place, what would have helped for you to do your job better? Are there aspects of the job you are doing better?
- If another service came to ask your advice about how to bring about these changes, what would you tell them/advise how best to make these changes?

## Semi-structured Interview Guide for children & young people:

### Changes/transformations (RE-AIM Framework – ‘RE’ focuses on service user)

#### Reach:

- Can you tell me a little bit about how you came to use CAMHS services?
- How did you access CAMHS? Can you describe the process you went through?
- Have you accessed CAMHS before? What did you know about CAMHS before you accessed it?
- How easy/difficult was it to get in touch with CAMHS?/
- Did you use the Single Point of Access?
- What was your experience of accessing CAMHS? (if negative, how do you think this could have been improved?)
- (If applicable), how did this compare to accessing it before?

#### Effectiveness:

- Were you aware of any of the new changes happening? What do you think of those changes (go through prompts – explain each one)?
- Before you accessed CAMHS what did you expect would happen?
- When you accessed CAMHS who was involved in your care – what were they like? What information did they give you? Did you find the information helpful? How could it be improved?
- What information did you need?/what would have been helpful to you?
- What has happened since you accessed CAMHS the first time?
- If you could imagine an ‘ideal’ service how would that look like?
- What do you think could have made the service improve/do better?
- If you had a message for CAMHS what would that be and why?
- If you had a message for other people using CAMHS what would that be?
